# Measuring and Managing Obesity in Pregnancy Using the Edmonton Obesity Staging System: A Scoping Review

**DOI:** 10.1111/cob.70043

**Published:** 2025-08-28

**Authors:** Taniya S. Nagpal, Jordyn M. Cox, Ximena Ramos Salas, Kristi B. Adamo

**Affiliations:** ^1^ Faculty of Kinesiology, Sport, and Recreation University of Alberta Edmonton Alberta Canada; ^2^ Bias 180, Ontario, Canada & Replica Communications Kristianstad Sweden; ^3^ School of Human Kinetics, University of Ottawa Ottawa Ontario Canada

**Keywords:** body mass index, Edmonton obesity staging system, healthcare, maternal care, obesity, pregnancy

## Abstract

Emerging evidence and clinical practice guidelines have highlighted that obesity, defined as a chronic disease characterised by excess or dysfunctional adipose tissue, may not be accurately measured or understood by solely relying on body mass index (BMI) which is a measure of size not functionality. An alternative to BMI, as proposed in the Canadian Adult Obesity Management Guideline, is the use of the Edmonton Obesity Staging System (EOSS). While the EOSS has been evaluated in both adult and paediatric populations, pregnant individuals remain an underrepresented clinical group in its application. Prenatal care relies on BMI for measurement of maternal obesity; however, the EOSS may be an adjunct or alternative method to consider. This scoping review aimed to summarise previous research on EOSS in pregnancy and to advise future directions. Only three cohort studies were identified, emphasising a critical gap in obesity research. Both BMI and higher EOSS stages (i.e., 3 and 4) were associated with prenatal complications (e.g., preeclampsia, venous thromboembolism, wound complications). Given that EOSS has been used in other populations and is noted to be an effective patient‐centred tool to diagnose and manage obesity, future work may explore its use in pregnancy both in comparison to and in conjunction with BMI.

## 1 Introduction

Obesity during pregnancy is associated with several adverse outcomes, including increased risk for maternal morbidities (e.g., gestational diabetes, preeclampsia and depression) and labour and delivery complications (e.g., preterm birth, shoulder dystocia, and vaginal tears) [1, 2, 3]. Accordingly, obesity and pregnancy guidelines suggest that individuals should aim to reduce their weight before conceiving to prevent complications [4]. Furthermore, throughout pregnancy, individuals with obesity may be referred to specialist care, which can include frequent foetal monitoring, early screening for gestational diabetes, and gestational weight management [5]. The risks associated with obesity in pregnancy are positively correlated with body mass index (BMI ≥ 30.0 kg/m^2^), such that individuals with a higher BMI are at greater risk of experiencing poor health outcomes [1, 3].

Obesity—a chronic disease characterised by dysfunctional or excess adipose tissue that impairs health and wellbeing—requires a more comprehensive measurement tool than BMI alone [6]. There is much criticism surrounding BMI alone as a clinical tool to diagnose obesity, given that it only considers one's height and weight and does not capture measures of individuals' functioning [7], adipose distribution and dysregulation, other obesity complications, and psychosocial implications [8, 9, 10]. In pregnancy, BMI guides clinical decision‐making in many situations, such as cut‐offs for gestational weight gain, screening for gestational diabetes, and referrals for specialist care [11]. Moreover, before pregnancy, BMI may be used to guide decision‐making surrounding preconception care, including eligibility for in vitro fertilisation and weight loss targets before trying to conceive [5]. Indeed, BMI is a population‐level assessment that can aid in understanding trends [12]. At the clinical level, BMI can be used as a screening tool to identify individuals who may be at risk for obesity and related complications. In fact, a recent review highlighted that BMI is usually sufficient to represent excess adiposity, but it cannot distinguish the severity of obesity and related complications on its own [13]. Although there is research supporting the association between maternal BMI and pregnancy complications, it may be prudent to consider alternative or adjunct options to better personalise care and for diagnosing obesity, especially for those who may require high‐risk obesity care in pregnancy [9, 12, 14]. In pregnancy, previous studies have reported that individuals who received high‐risk care solely based on their BMI with the absence of an obesity diagnosis or related complications felt increased stress throughout gestation as they anticipated negative outcomes, a lack of personalised advice for health behaviours like physical activity and nutrition, and poor communication with their healthcare provider [15]. Consequently, relying only on BMI to guide prenatal care may prevent establishing the patient‐centred approach necessary for effective obesity management. The Edmonton Obesity Staging System (EOSS), which includes assessment of BMI, offers a simple staging framework to guide clinical decision‐making for obesity that takes into account the impact of dysfunctional or excess adiposity on physical, metabolic, mental, and social health [9].

The EOSS has four stages and uses BMI as a risk factor in all stages [9]. Stage 0 includes an elevated BMI without additional obesity‐related risk factors (e.g., high blood pressure), and no limitations on functionality or well‐being. Stages 1 to 4 then progressively increase in terms of health impairments and obesity complications. Stage 1 includes subclinical markers (e.g., insulin resistance) that can be managed by behavioural interventions and mild limitations to functionality and well‐being. Stage 2 refers to established obesity‐related complications (e.g., musculskeletal disorders, type 2 diabetes) and moderate limitations to functionality and well‐being, which may require both behavioural interventions and other adjunct medical interventions. Stage 3 indicates organ damage, and finally, Stage 4 is disabling complications, resulting in significant to severe functional limitations and poor well‐being. In Stages 3 and 4, greater medical interventions plus behavioural interventions are deemed necessary. At Stage 0, BMI is a risk factor that could indicate a potential increase in EOSS stages in the future. At the later EOSS stages, BMI remains an embedded feature and is assessed in conjunction with other physical, functional, and social variables that may be implicated by excess or dysfunctional adiposity. Several studies that have used EOSS to stage obesity have noted that it is a significant predictor for critical obesity‐related outcomes like mortality, postoperative complications, resolution of hypertension, and quality of life, as well as an effective tool to guide patient‐centred conversations about obesity risk and severity in comparison to BMI alone [16, 17, 18, 19].

In addition to EOSS, other frameworks have been proposed such as the King's Obesity Staging System (KOSC), which uses nine domains to classify patients into severity levels of obesity [20]. Similar to EOSS, the KOSC assesses functional limitations as a result of obesity, but is also more specifically focused on cardiovascular risk and diabetes as key determinants of severity [20, 21]. There are several alternate anthropometric measures that are recommended other than BMI including using waist circumference and waist‐to‐hip ratio, which have been associated with better predictability of cardiovascular disease [22, 23]. Rather than relying solely on BMI cut‐offs, percent body fat provides greater specificity in identifying obesity‐related complications, especially amongst racially diverse populations [24]. As a result, adiposity measures are recommended over the traditional use of height and weight alone. Several health organisations, including the American Association of Clinical Endocrinology, advocate for adopting the use of Adiposity Based Chronic Disease or ABCD instead of obesity to better characterise and evaluate the condition [25]. Additionally, international guidelines and expert consensus statements recommend that as a chronic, relapsing disease, in addition to a clinical diagnosis, obesity should be clinically staged according to the severity of its medical, mental, and functional complications [6, 26, 27]. The EOSS is recognised as a simple tool to evaluate and describe an individual's health status and to establish obesity risk and severity, and most recently, was recommended as a staging tool in the Canadian Adult Obesity Clinical Practice Guideline [6]; this guideline is recognised internationally as it provides a comprehensive overview of obesity as a chronic disease and its management, and it has been adapted to several other countries such as Netherlands, Chile, Ireland, Mexico, and Spain. While still acknowledging there are other suggested clinical staging systems for obesity, EOSS is being considered in the present review given its recent international recognition in clinical practice guidelines and positive findings in terms of systematically evaluating medical, functional, and psychological impairments of obesity, determining the progression of obesity, guiding personalised obesity treatment and management interventions, and establishing patient‐provider rapport in non‐pregnant populations (note: EOSS was not included in current pregnancy chapters in the Canadian or adapted guidelines).

The EOSS framework, designed for adults, has been adapted for specific population groups such as paediatrics and adolescents, and has consistently been noted to be effective in tailoring patient care for obesity [19, 28]. Accordingly, the EOSS may also be a useful framework to understand obesity in pregnancy and inform improvement in the quality of care. The purpose of this scoping review was to summarise the literature examining EOSS use in pregnancy. Scoping review methods were selected because they enable a broad examination of the literature, offering a comprehensive overview of a novel topic to identify knowledge gaps and guide future research directions [29].

## 2 Methods

This scoping review was completed following the 2020 Preferred Reporting Items for Systematic Reviews and Meta‐Analyses extension for scoping reviews (checklist available in Table A1 of Appendix) [29]. The main objective of this review was to map the existing literature on the use of EOSS in pregnancy, including assessing any outcomes and comparisons with BMI alone.

### 2.1 Search Strategy

A search strategy was developed with the keywords ‘pregnancy’ and ‘Edmonton Obesity Staging System’ (search strategy for Medline presented in Table 1) and checked by an information specialist. The following databases were screened, and the search strategy was modified accordingly: Medline, Web of Science and Proquest Thesis and Dissertations. The search was conducted on 7 August 2023, without imposing date or language restrictions.

**TABLE 1:** Search for Medline.

Keyword	Search string	Articles retrieved
Pregnant	(pregnancy) OR (prenatal) OR (perinatal) OR (gestation) OR (pregnant)	1 286 454
Edmonton obesity staging system	(Edmonton Obesity Staging System) OR (EOSS)	2558
Total retrieved	Above searches added with AND	183

### 2.2 Inclusion and Exclusion Criteria

To be included, studies needed to meet these criteria: 1. Pregnant population; 2. Use of the Edmonton Obesity Staging System (i.e., to identify severity of obesity; to determine obesity treatment and long term‐management; or to determine prenatal care). All study designs, including qualitative investigations, cross‐sectional and cohort studies, and controlled trials, were considered eligible. Published abstracts were eligible if all other criteria were met as per scoping review guidelines. Reviews, editorials, commentaries, protocols, animal studies, and opinion papers were excluded; however, their reference lists were screened to identify any other potential eligible articles.

### 2.3 Screening Process

Retrieved articles were transferred to Endnote software and the automatic duplicate removal feature was used. The remaining articles were uploaded to Covidence software for further screening. Two independent reviewers screened all titles/abstracts to identify potentially relevant articles. Following this stage, full texts were screened to confirm eligibility. If there were any discrepancies, a third reviewer was consulted.

### 2.4 Data Extraction

Data from included studies were extracted into a standardised Excel spreadsheet. The following data were extracted from each paper: country of study, year published, study design, number of participants, and participant pool description (i.e., average age, pre‐pregnancy BMI), and overview of methods. The main findings were recorded in relation to EOSS and if there was any comparison to BMI. Finally, we also extracted if authors had suggested any modification to the original EOSS framework for use in pregnancy. All extractions were completed by one reviewer and checked in full by a second reviewer. Scoping review methods are different from systematic reviews such that the primary objective is not a synthesis; instead, authors may be striving to identify knowledge gaps, generally summarise the scope of the literature on a specific and novel topic, or clarify concepts [30]. Scoping reviews can help determine whether there is sufficient research available to conduct a systematic review or guide early studies, especially for new or under‐researched topics. Accordingly, the present study adheres to scoping review expectations, and in particular, extracted data were summarised to provide a general scope of the existing literature on EOSS in pregnancy and identify knowledge gaps.

### 2.5 Data Mapping

Data were summarised narratively such that all findings are presented as an overview of what has been previously done and reported in regard to the use of EOSS in pregnancy. We established two main themes to describe the findings, including outcomes that have been assessed using EOSS in pregnancy and comparisons between EOSS and BMI alone.

## 3 Results

**FIGURE 1:**
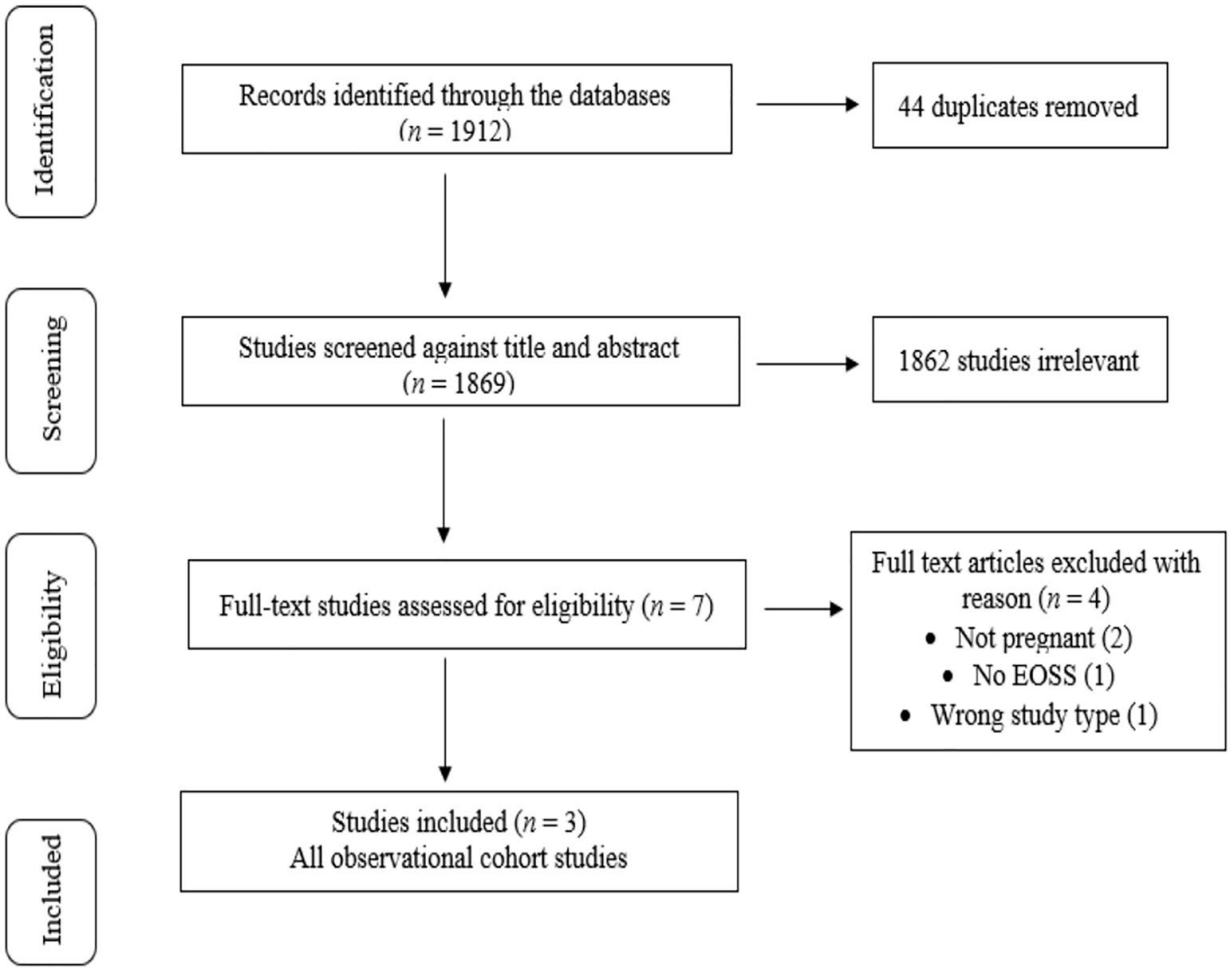
PRISMA flow diagram.

The search resulted in 1913 articles retrieved, and 3 were included in the review (Figure 1). The majority of papers that were excluded at titles/abstracts did not include pregnant populations. One study was conducted in Canada, one was in Ireland, and one in the United States [31, 32, 33]. One was a prospective cohort study, and two were retrospective cohort studies. Table 2 lists study characteristics, a description of methods, and main findings specifically in relation to EOSS. One study was presented as an abstract only, and a full paper was not retrieved in our search [33]. One study made modifications to the original EOSS for pregnancy, including removing questions that would likely be irrelevant to reproductive‐age females (e.g., liver failure), adding questions specific to obstetric and gynaecological comorbidities related to obesity, and fewer questions on mental health and mortality to avoid increasing fear or stress [31].

**TABLE 2:** Study characteristics and findings specific to using the Edmonton Obesity Staging System (EOSS) in pregnancy.

Author, year, country	Sample	Type of study	Describe study methodology	Main findings pertaining to EOSS and pregnancy	Is EOSS compared to BMI?
Demsky, 2020, Canada	*N* = 345 Age: Control group = 30.0 median Cohort group = 29.5 median BMI: Control group = 27.0 median Cohort group = 34.3 median	Prospective cohort	Participants completed a personal health history. They were categorised into two groups based on their measured weight at the first antenatal visit: A sample cohort of women with a BMI ≥ 25 and a control cohort of women with a BMI of 18.5 to 24.9. Before delivery, researchers independently assigned women in the sample cohort to different EOSS categories based on their obesity‐related comorbidities. The most severe comorbidity determined the EOSS category for patients with multiple comorbidities. Variables were gathered from self‐reported data or from hospital records.	Found that the EOSS may help predict the chance of caesarean delivery in a high‐risk group of nulliparous women with overweight or obesity who are undergoing an induction of labour at term.	EOSS level 3 and above significantly predicted C‐section risk. Although BMI and C‐section risk was also positively correlated, this did not reach significance.
Killeen, 2022, Ireland	*N* = 348 Age: 32.4 (4.4) BMI: 28.0 (range = 26.6–29.9)	Retrospective cohort	Assessed patient records to evaluate the relationship between pregnancy outcomes and EOSS, and fasting lipids and glucose were measured in early (14–16 weeks) and late (28 weeks) pregnancy in women with a BMI ≥ 25. Blood pressure was collected from medical records, and maternal well‐being was evaluated using the World Health Organization‐5 Well‐being Index.	Found that most women with a high BMI had a high EOSS score, but these scores did not reliably predict pregnancy outcomes. Increased EOSS scores were primarily influenced by elevated total cholesterol, potentially limiting its effectiveness in predicting adverse outcomes.	No.
White, 2022, USA	*N* = 169 433 Age: Not reported BMI: Not reported	Retrospective cohort	Categorised patients with obesity using chart data with a modified EOSS for pregnancy. The primary outcome was a composite of maternal adverse events, including chorioamnionitis, endometritis, preeclampsia with severe features, eclampsia, venous thromboembolism, wound complication, intensive care unit admission, and maternal death.	EOSS stages were associated with increased rates of all defined adverse outcomes compared to the individuals who did not have obesity using.	Both BMI and EOSS were comparable across each outcome assessed, showing positive associations.

Abbreviations: BMI, body mass index; EOSS, Edmonton Obesity Staging System.

### 3.1 Outcomes Assessed

The following outcomes were assessed: C‐section, birthweight, gestational age at delivery, preeclampsia, chorioamnionitis, endometritis, venous thromboembolism, wound complication, intensive care unit admission, maternal death and pregnancy after fertility treatment. In one prospective cohort study, participants with a BMI ≥ 25.0 kg/m^2^ completed a personal health history questionnaire which authors then used to categorise into the EOSS stages [31]. Outcomes were assessed using hospital chart data, and it was found that greater EOSS stage was associated with C‐section delivery amongst high‐risk nulliparous participants who had overweight or obesity and were induced for labour [31]. Notably, Demsky et al. (2020) was the only included study that modified the original EOSS based on expert opinion specifically for pregnancy. The modifications included adding obstetric and gynaecological complications that could be related to obesity in the assessment of comorbidities (e.g., preeclampsia, diabetes complications), as well as removing questions as described above that may be considered irrelevant in the context of pregnancy. Although the authors found an increased risk of C‐section deliveries with EOSS Stage 3 and above, it should also be noted that many of the pregnancy morbidities and chronic conditions associated with obesity and organ damage (e.g., renal insufficiency, myocardial infraction, angina) may also result in labour and delivery complications. Killeen et al. (2022) did not find a significant association between EOSS stage and C‐section delivery and other outcomes including fasting lipids and glucose in early (14–18 weeks) and mid‐ to late pregnancy (28 weeks), birthweight and gestational age at delivery [32]. Authors reported that in their retrospective cohort, which was a secondary analysis from a previous intervention trial, most participants had EOSS scores above Stage 0 mostly due to elevated cholesterol; however, few were in Stages 3 or 4 where there may have been significant associations with pregnancy complications. Furthermore, Killeen et al. (2022) focused on staging obesity using the EOSS considering metabolic implications, not physical or functional limitations as is described in clinical practice guidelines for obesity management. The authors conclude that with the available data and findings, the clinical utility of EOSS in pregnancy is limited. White et al. (2022) completed a retrospective cohort study and found that EOSS stage was correlated with several critical outcomes including chorioamnionitis, endometritis, preeclampsia, eclampsia, venous thromboembolism, wound complication, intensive care unit admission, and maternal death [33]. However, having access to only a published abstract for the White et al. (2022) study, these results should be interpreted with caution given that there is no available information on any modifications made to the EOSS or how it was used.

### 3.2 EOSS and BMI

Demsky et al. (2020) found that the rate of C‐sections was correlated with BMI alone; however, this relationship did not reach statistical significance, whereas the correlation with EOSS stage did [31]. When assessed cross‐sectionally, EOSS Stages 0 to 2 did not significantly increase the risk for C‐section deliveries. However, later stages of EOSS, which consider several prenatal and obesity comorbidities (e.g., preeclampsia, diabetes complications, pulmonary oedema) could elevate the risk for labour and delivery complications. Further, BMI alone also showed a non‐significant stepwise increase in the risk of C‐sections. Therefore, no conclusive statement can be made about EOSS versus BMI alone regarding C‐sections, as both metrics appear to be positively correlated with this delivery mode. Similarly, White et al. (2022) report that both high BMI and higher EOSS levels are associated with greater pregnancy complications, but no conclusion can be drawn with the available data regarding their predictive capabilities [33]. Overall, available evidence does not suggest superiority of EOSS or BMI use in pregnancy. None of the included studies addressed the patient‐centredness of using EOSS in comparison to, or in conjunction with, BMI.

## 4 Discussion

This scoping review summarised the limited literature on EOSS use during pregnancy. The data available indicate that similar to BMI, higher EOSS stages in pregnancy are associated with greater prenatal complications. For example, EOSS Stages 3 and 4 were associated with C‐section, chorioamnionitis, endometritis, preeclampsia, eclampsia, venous thromboembolism, wound complication, intensive care unit admission, and maternal death. Accordingly, available evidence does not allow for drawing any conclusions regarding the use of EOSS versus BMI in pregnancy. This scoping review identified that there is a paucity of data available on the clinical utility of EOSS in pregnancy, and there is no evidence to suggest that it is superior to BMI in its predictive value regarding complication risk. Moreover, unlike studies with non‐pregnant populations, none of the included studies assessed the potential impact of EOSS in improving patient‐centred and non‐stigmatising obesity care, and this is an important avenue for further research. These limited findings highlight the need for further research on the use of EOSS alongside or compared to BMI alone in pregnancy before any clinical recommendations can be made.

Previous research on obesity in pregnancy has almost entirely relied on BMI alone. In fact, several studies have reported associations between BMI and critical prenatal outcomes including pregnancy loss, congenital abnormalities, shoulder dystocia, placental deficiencies, venous thromboembolism postpartum, and excessive gestational weight gain [3, 34, 35]. Although using BMI as a population level tool is well accepted [12], there may be an unintended consequence of increased stigma when affirmative claims are made on the relationship between high BMI (a measure of height and weight) and negative prenatal health outcomes. Individuals with larger bodies who are pregnant have reported feeling ‘blamed’ and ‘guilty’ for causing potential harm to their child even when no complications are present [15]. These feelings of blame and guilt can be internalised by individuals, impacting their physical and mental health outcomes [36, 37]. Moreover, media outlets have taken research findings that have shown associations between BMI and significant negative health outcomes, like maternal death, and portrayed them as a crisis for which the individual with a high BMI is personally responsible [38]. In the media, associations with BMI are presented as causal, and this can lead to misunderstandings amongst the public that all individuals with larger bodies are susceptible to detrimental maternal and newborn complications [39]. Furthermore, the simplification of obesity in pregnancy to a behavioural issue rather than as a complex chronic disease can also be a barrier for access to evidence‐based and effective obesity care [38]. There is some research that indicates that the belief that obesity is a behavioural issue can lead to pregnant individuals receiving negative comments about their nutrition and physical activity patterns, which increases the risk for prenatal and postpartum depression [40]. Accordingly, health claims made using BMI alone need to include education surrounding its limitations to avoid unwarranted harm like weight stigma [41].

Excess adiposity, even without other functional or social limitations, can increase the risk of birth complications like infections from deep wound incisions after a C‐section delivery [42], and reduced placental efficiency [43]. Therefore, BMI at this time should remain a staple of prenatal care, but healthcare providers should acknowledge its limitations given that it is not a comprehensive assessment of obesity. Accordingly, it may be helpful to test a staging system like EOSS that accounts for BMI, but also incorporates other obesity‐related physical (e.g., musculoskeletal disorders), metabolic (e.g., insulin resistance), mental (e.g., depressive symptoms) and social (e.g., social withdrawal) impairments that can cause negative prenatal experiences and outcomes. Accordingly, it may be necessary to consider adapting/modifying EOSS from its original form to be more specific to pregnancy considering prenatal‐specific changes to health (e.g., gestational weight gain, gestational diabetes); only one study made a modification to EOSS for prenatal use [31], but findings were not assessed in a way to confirm if the modifications were indeed helpful in improving the function of EOSS in pregnancy. Future research may investigate the prenatal experience of receiving obesity care based on body size alone (i.e., BMI) versus the use of a more comprehensive obesity assessment and classification framework, such as the EOSS. Overall, there is a need to ensure that the delivery of prenatal care is sensitive, non‐stigmatising, and patient‐centred to improve the quality of care and maternal and newborn outcomes. Of note, no studies have assessed whether EOSS promoted patient‐centredness, including in comparison to BMI. While evidence from non‐pregnant populations suggests that EOSS has the potential to enhance patient‐centred care, its effectiveness during pregnancy remains unknown and represents another future direction for research.

Current data suggest that EOSS Stages 3 and 4 predict complications. Using EOSS early in pregnancy could help identify high‐risk patients and provide preventive care sooner. Patients with a BMI in the 30.0–35.0 kg/m^2^ range have reported feeling fearful and anxious about complications that never occurred due to early BMI‐based discussions [15]. A prospective study establishing EOSS stage early in pregnancy and evaluation of downstream complications in comparison to BMI alone may help inform whether EOSS should be used to better classify patients who may need high‐risk prenatal care. Regarding BMI, although most guidelines have suggested that a cut‐off of 40.0 kg/m^2^ is likely more useful to establish risk profiles than 30.0–39.9 kg/m^2^, several studies have still shown risk of complications associated with lower BMI cut‐offs and this may be because BMI does not capture other indices of health considering metabolic profiles or psychosocial variables.

An argument in favour of using BMI over other assessment and obesity classification systems, such as DXA (dual x‐ray absorptiometry) in non‐pregnant adult populations that captures distribution of adiposity, is its feasibility [14]. However, EOSS is also a low‐cost, valid staging tool that could facilitate patient‐centred conversations about the multifaceted causes of obesity and barriers to management [44]. Although promising, its application and impact during pregnancy remains to be tested. To use EOSS, a healthcare provider needs to perform a patient history, standardised measures if needed (e.g., blood test), and administer an interview or questionnaires on well‐being [6, 9]. Additional tests may be required depending on co‐morbidities [6]. These measures allow a healthcare provider to more holistically understand the patient's obesity, its associations with markers of health other than weight or body size alone, and impact on the life of a patient. Previous studies with non‐pregnant populations have shown that EOSS is feasible to use in clinical settings and can allow for better patient‐physician rapport than using BMI alone to discuss obesity and offer management options beyond focusing on just weight loss [9, 28, 44]. Furthermore, EOSS may be translated to a self‐report questionnaire if necessary, which further increases its accessibility and this has shown good internal validity [45]. Future research should aim to assess the feasibility of using EOSS prospectively in prenatal clinical settings, including its cost‐effectiveness. The validity of applying the EOSS during pregnancy must be assessed and established before it can be integrated into standard care.

The studies included in this review used cohort methods and tested correlations between EOSS, BMI and outcomes, which do not establish causation. Therefore, a future research direction may also be to test EOSS, as well as other frameworks that have been proposed to measure obesity, such as the KOSC or the newly proposed European Association for the Study of Obesity staging system, in comparison to or in conjunction with BMI in clinical trials and prospective cohorts to better establish relationships with health outcomes, test clinical superiority, and patient‐provider rapport, and inform which tool would be most appropriate to use in pregnancy. It should be observed that several obesity and pregnancy clinical practice guidelines recommend the use of BMI to guide care (e.g., determining the need for transfer of care to high risk clinics, earlier testing for gestational diabetes, guidelines for gestational weight gain) [4, 46, 47, 48]. The positive relationship between BMI and pregnancy complications has been consistently reported (e.g., C‐section delivery, hypertensive disorders, gestational diabetes) [49]. There is not enough evidence to suggest BMI should not be utilised in pregnancy care, but healthcare professionals should acknowledge, in current international obesity diagnosis, staging, and management clinical practice guidelines, that it is not considered a comprehensive obesity diagnostic tool. The EOSS has been widely tested in non‐pregnant adult populations, but it is also not considered standard of practice across all regions and countries and requires further testing amongst racially and ethnically diverse groups. At this time, with the current findings, we do not recommend replacing BMI with EOSS in prenatal care but do suggest that further prospective investigations are warranted, including evaluating if the EOSS in pregnancy does impact quality of care, such as improving patient‐provider rapport.

This is the first review that has aimed to summarise the literature on EOSS use in pregnancy, and limited results were retrieved, highlighting a knowledge gap. Findings were summarised individually given the limited data available, and the discussion offers suggestions for further research. Additional strengths include following the PRISMA‐ScR to guide the search and data mapping. Limitations of this review are the few identified studies and as we used scoping review methods, in which study quality is not usually considered. Due to the limited number of studies in this area, we provided a summary of study quality using the descriptive Mixed Methods Appraisal Tool for cohort studies (Table S1) [50]. Of the three identified, only two could be assessed given one was available only in abstract form. Although the two did appear to meet the key criteria for cohort studies, the overall scarcity of data suggests that a systematic review and meta‐analysis would not be recommended at this time.

## 5 Conclusion

This scoping review identified three cohort studies that have used the EOSS in pregnancy. Similar to BMI, EOSS stage is correlated with prenatal complications. No study has assessed whether or not EOSS improves the delivery of patient‐centred obesity care during pregnancy. Further research is needed to test the use of EOSS in pregnancy to guide clinical decision‐making, its impact on patient‐centred care, and establish its feasibility and effectiveness, especially in comparison with current standards that primarily rely on BMI alone.

## Author Contributions

T.S.N., K.B.A. and X.R.S. conceptualised the review. T.S.N. and J.M.C. conducted the search. J.M.C. led data screening and extraction. All authors contributed to data interpretation. T.S.N. drafted the manuscript. J.M.C. drafted the tables and figures. All authors edited and approved the final submission.

## Conflicts of Interest

The authors declare no conflicts of interest.

## Supporting information


**Table S1:** Quality appraisal of included studies: Quantitative non‐randomised studies mixed methods appraisal tool (2018).

## Data Availability

Data sharing is not applicable to this article as no new data were created or analysed in this study.

## Appendix

**TABLE A1:** PRISMA‐ScR checklist item.

Section	Item	PRISMA‐ScR checklist item	Reported on page number
Title			
Title	1	Identify the report as a scoping review.	1
Abstract			
Structured summary	2	Provide a structured summary that includes (as applicable): background, objectives, eligibility criteria, sources of evidence, charting methods, results, and conclusions that relate to the review questions and objectives.	2
Introduction			
Rationale	3	Describe the rationale for the review in the context of what is already known. Explain why the review questions/objectives lend themselves to a scoping review approach.	3–6
Objectives	4	Provide an explicit statement of the questions and objectives being addressed with reference to their key elements (e.g., population or participants, concepts, and context) or other relevant key elements used to conceptualise the review questions and/or objectives.	6
Methods			
Protocol and registration	5	Indicate whether a review protocol exists; state if and where it can be accessed (e.g., a Web address); and if available, provide registration information, including the registration number.	7
Eligibility criteria	6	Specify characteristics of the sources of evidence used as eligibility criteria (e.g., years considered, language, and publication status), and provide a rationale.	7
Information sources^a^	7	Describe all information sources in the search (e.g., databases with dates of coverage and contact with authors to identify additional sources), as well as the date the most recent search was executed.	7
Search	8	Present the full electronic search strategy for at least 1 database, including any limits used, such that it could be repeated.	Table 1
Selection of sources of evidence^b^	9	State the process for selecting sources of evidence (i.e., screening and eligibility) included in the scoping review.	7–8
Data charting process^c^	10	Describe the methods of charting data from the included sources of evidence (e.g., calibrated forms or forms that have been tested by the team before their use, and whether data charting was done independently or in duplicate) and any processes for obtaining and confirming data from investigators.	8
Data items	11	List and define all variables for which data were sought and any assumptions and simplifications made.	7–8
Critical appraisal of individual sources of evidence^d^	12	If done, provide a rationale for conducting a critical appraisal of included sources of evidence; describe the methods used and how this information was used in any data synthesis (if appropriate).	16
Synthesis of results	13	Describe the methods of handling and summarising the data that were charted.	8
Results			
Selection of sources of evidence	14	Give numbers of sources of evidence screened, assessed for eligibility, and included in the review, with reasons for exclusions at each stage, ideally using a flow diagram.	9
Characteristics of sources of evidence	15	For each source of evidence, present characteristics for which data were charted and provide the citations.	9
Critical appraisal within sources of evidence	16	If done, present data on critical appraisal of included sources of evidence (see item 12).	16
Results of individual sources of evidence	17	For each included source of evidence, present the relevant data that were charted that relate to the review questions and objectives.	9–11
Synthesis of results	18	Summarise and/or present the charting results as they relate to the review questions and objectives.	9–11
Discussion			
Summary of evidence	19	Summarise the main results (including an overview of concepts, themes, and types of evidence available), link to the review questions and objectives, and consider the relevance to key groups.	11–12
Limitations	20	Discuss the limitations of the scoping review process.	16
Conclusions	21	Provide a general interpretation of the results with respect to the review questions and objectives, as well as potential implications and/or next steps.	17
Funding			
Funding	22	Describe sources of funding for the included sources of evidence, as well as sources of funding for the scoping review. Describe the role of the funders of the scoping review.	NA

Abbreviations: JBI = Joanna Briggs Institute; PRISMA‐ScR = Preferred Reporting Items for Systematic reviews and Meta‐Analyses extension for Scoping Reviews.

^a^ Where *sources of evidence* (see second footnote) are compiled from, such as bibliographic databases, social media platforms, and Web sites.

^b^ A more inclusive/heterogeneous term used to account for the different types of evidence or data sources (e.g., quantitative and/or qualitative research, expert opinion, and policy documents) that may be eligible in a scoping review as opposed to only studies. This is not to be confused with *information sources* (see first footnote).

^c^ The frameworks by Arksey and O'Malley and Levac and colleagues and the JBI guidance [4] refer to the process of data extraction in a scoping review as data charting.

^d^ The process of systematically examining research evidence to assess its validity, results, and relevance before using it to inform a decision. This term is used for items 12 and 19 instead of ‘risk of bias’ (which is more applicable to systematic reviews of interventions) to include and acknowledge the various sources of evidence that may be used in a scoping review (e.g., quantitative and/or qualitative research, expert opinion, and policy document).

*From*: Tricco et al. [29].
