# Supplementary material for: Measuring and Managing Obesity in Pregnancy Using the Edmonton Obesity Staging System: A Scoping Review
Source: Clin Obes. 2025 Aug 28;16(1):e70043. doi: 10.1111/cob.70043 (PMC12705254; doi:10.1111/cob.70043)
Supplement: Supplementary file 1 — Table S1: Quality appraisal of included studies: Quantitative non‐randomised studies mixed methods appraisal tool (2018). [file COB-16-e70043-s001.docx]

**Supplementary Table 1:** Quality appraisal of included studies: Quantitative Non-randomized studies Mixed Methods Appraisal Tool (2018)

| **Study** | **Methodological quality criteria** | **Yes** | **No** | **Can’t Tell** | **Comments** |
| --- | --- | --- | --- | --- | --- |
| Demsky, A. N., Stafford, S. M., Birch, D., Sharma, A. M., Schulz, J. A., & Steed, H. (2020). The Edmonton Obesity Staging System predicts mode of delivery after labour induction. *Journal of Obstetrics and Gynaecology Canada*, *42*(3), 284-292. | 3.1. Are the participants representative of the target population? | x |  |  |  |
|  | 3.2. Are measurements appropriate regarding both the outcome and intervention (or exposure)? | X |  |  |  |
|  | 3.3. Are there complete outcome data? | X |  |  |  |
|  | 3.4. Are the confounders accounted for in the design and analysis? | x |  |  |  |
|  | 3.5. During the study period, is the intervention administered (or exposure occurred) as intended? | X |  |  |  |
| Killeen, S. L., Yelverton, C. A., Geraghty, A. A., Kennelly, M. A., Eakins, S., Farrell, L., ... & McAuliffe, F. M. (2022). The Edmonton Obesity Staging System and pregnancy outcomes in women with overweight or obesity: A secondary analysis of a randomized controlled trial. *Clinical Obesity*, *12*(3), e12510. | 3.1. Are the participants representative of the target population? | X |  |  |  |
|  | 3.2. Are measurements appropriate regarding both the outcome and intervention (or exposure)? | X |  |  |  |
|  | 3.3. Are there complete outcome data? | X |  |  |  |
|  | 3.4. Are the confounders accounted for in the design and analysis? | X |  |  |  |
|  | 3.5. During the study period, is the intervention administered (or exposure occurred) as intended? | x |  |  | Secondary analysis. |
| White S, Stamilio DM. The use of the Edmonton Obesity Staging System to predict adverse pregnancy outcomes. *American Journal of Obstetrics & Gynecology*. 2022;226(1):S577-S578 | 3.1. Are the participants representative of the target population? |  |  | x | Abstract |
|  | 3.2. Are measurements appropriate regarding both the outcome and intervention (or exposure)? |  |  | x |  |
|  | 3.3. Are there complete outcome data? |  |  | x |  |
|  | 3.4. Are the confounders accounted for in the design and analysis? |  |  | x |  |
|  | 3.5. During the study period, is the intervention administered (or exposure occurred) as intended? |  |  | x |  |

Reference: Hong, Quan Nha, Pierre Pluye, Sergi Fàbregues, Gillian Bartlett, Felicity Boardman, Margaret Cargo, Pierre Dagenais et al. "Mixed methods appraisal tool (MMAT), version 2018." *Registration of copyright* 1148552, no. 10 (2018): 1-7.
